# Electrocatalytic Properties of Co_3_O_4_ Prepared on Carbon Fibers by Thermal Metal–Organic Deposition for the Oxygen Evolution Reaction in Alkaline Water Electrolysis

**DOI:** 10.3390/nano13061021

**Published:** 2023-03-12

**Authors:** Myeong Gyu Kim, Yun-Hyuk Choi

**Affiliations:** School of Advanced Materials and Chemical Engineering, Daegu Catholic University, Gyeongsan 38430, Republic of Korea

**Keywords:** Co_3_O_4_, metal–organic deposition, electrocatalyst, oxygen evolution reaction, alkaline water electrolysis

## Abstract

Cobalt oxide (Co_3_O_4_) serves as a promising electrocatalyst for oxygen evolution reactions (OER) in water-electrolytic hydrogen production. For more practical applications, advances in dry-deposition processes for the high-throughput fabrication of such Co_3_O_4_ electrocatalysts are needed. In this work, a thermal metal–organic deposition (MOD) technique is developed to form Co_3_O_4_ deposits on microscale-diameter carbon fibers constituting a carbon fiber paper (CFP) substrate for high-efficiency OER electrocatalyst applications. The Co_3_O_4_ electrocatalysts are deposited while uniformly covering the surface of individual carbon fibers in the reaction temperature range from 400 to 800 °C under an ambient Ar atmosphere. It is found that the microstructure of deposits is dependent on the reaction temperature. The Co_3_O_4_ electrocatalysts prepared at 500 °C and over exhibit values of 355–384 mV in overpotential (η_10_) required to reach a current density of 10 mA cm^−2^ and 70–79 mV dec^−1^ in Tafel slope, measured in 1 M KOH aqueous solution. As a result, it is highlighted that the improved crystallinity of the Co_3_O_4_ electrocatalyst with the increased reaction temperature leads to an enhancement in electrode-level OER activity with the high electrochemically active surface area (ECSA), low charge transfer resistance (R_ct_), and low η_10_, due to the enhanced electrical conductivity. On the other hand, it is found that the inherent catalytic activity of the surface sites of the Co_3_O_4_, represented by the turnover frequency (TOF), decreases with reaction temperature due to the high-temperature sintering effect. This work provides the groundwork for the high-throughput fabrication and rational design of high-performance electrocatalysts.

## 1. Introduction

Demand for sustainable and renewable energy supply has surged as a solution to global warming in the 21st century [1,2]. Lots of studies have been conducted to utilize the so-called green energies such as solar, wind, waste heat, and hydrogen, replacing fossil fuels [2,3,4,5]. Among several recent successful attempts, the deployment of hydrogen energy has gained much attention, especially in green hydrogen production by electrochemical water electrolysis and the conversion of chemical energy into electricity using fuel cells [6]. One of the most important material parts in such systems is an electrocatalyst, which is composed of high-cost materials such as platinum and iridium oxide. At this point, since the high prices for those materials limits the extensive industrial applications of eco-friendly systems using hydrogen, the development of inexpensive, highly efficient, and durable electrocatalysts has intensively progressed [7,8,9].

The water electrolysis method can be classified into three methods: alkaline electrolysis, proton exchange membrane electrolysis, and solid oxide electrolysis depending on the type of electrolyte used. Among them, the alkaline electrolysis method is provided to supply 20–30 wt% of an electrolyte such as potassium hydroxide (KOH) and sodium hydroxide (NaOH) to a stack, and to produce hydrogen on a cathode and oxygen on an anode in a molar ratio of 2-to-1 through an electrochemical reaction. It completes the entire circuit by the transport of hydroxide anions (OH^−^) through an alkaline electrolyte, which is the most commercially available method because of its advantage of low system costs compared to other methods.

The electrochemical water electrolysis (2H_2_O → O_2_ + 2H_2_, E° = −1.23 V; ΔG = 475 kJ/mol) consists of two half-reactions: the hydrogen evolution reaction (HER, 2H^+^ + 2e^−^ → H_2_, E° = 0.00 V versus the normal hydrogen electrode (NHE)) at the cathode and the oxygen evolution reaction (OER, 2H_2_O → O_2_ + 4H^+^ + 4e^−^, E° = 1.23 V versus NHE) at the anode, where the overall reaction kinetics is dominated by the OER that four electrons are involved in [7,10]. Therefore, an appropriate catalyst is necessary to accelerate the OER at a low overpotential (η) for enhanced energy conversion efficiencies. State-of-the-art OER catalysts are based on precious metals such as IrO_2_ and RuO_2_ with the η values from 200 mV (in acid) to 300 mV (in base), but the scarcity and high costs of those materials restrict their large-scale applications [7]. In this regard, the earth-abundant transition metal oxides based on Co, Ni, Fe, and V have been extensively investigated as alternative OER catalysts [8,9,10,11,12,13,14,15].

Among various promising electrocatalytic materials, spinel-type Co_3_O_4_ has attracted attention in various fields such as methane combustion, CO oxidation, batteries, supercapacitors, and gas sensors due to its earth abundance, low cost, high catalytic activity, excellent redox property, and stability [16,17,18,19]. Although Co_3_O_4_ and related compounds exhibit particularly excellent inherent catalytic activity toward the OER for water electrolysis, their bulk forms show somewhat inferior electrode-level catalytic activity owing to their confined active surface area [20]. In this regard, intensive studies have focused on developing various methods (mainly using wet processing) that allow the nanostructuring of Co_3_O_4_ electrocatalysts to improve their OER activity by increasing the surface area and active site [20,21]. However, further investigation is required for the more industrially available dry-processing method in addition to the mechanistic study of electrocatalysis.

In this work, a thermal metal–organic deposition (MOD) technique is developed to form Co_3_O_4_ deposits on carbon fibers for high-efficiency OER electrocatalyst applications. The merit of this method is that the process operates at ambient pressure, where it enables more facile and cost-effective processing. Meanwhile, a thermal evaporation technique has been conventionally used to deposit oxide films in industrial fields, which is similar to this method but is clearly a different process. It requires a high-level vacuum and can easily form dense films with low catalytic activity, despite its high-throughput processing [22,23]. Therefore, the deposition of Co_3_O_4_ electrocatalysts on a paper-like substrate consisting of microscale-diameter carbon fibers by the thermal MOD is suggested as a promising process for the high-throughput fabrication of surface-dominated nanostructure electrocatalysts. Herein, we investigate the detailed electrocatalytic OER properties of the prepared Co_3_O_4_ electrocatalysts in an alkaline medium. This work contributes potentially to the large-scale, high-throughput production of catalytic electrodes via this unique preparative process.

## 2. Experimental

### 2.1. Preparation of Cobalt Oxide Electrocatalysts

The thermal metal–organic deposition (MOD) processes were performed using a horizontal cold-wall quartz tube (32 mm in diameter and 600 mm in length) furnace, which is equipped with gas flow controls. The furnace length was 400 mm, and both ends of the quartz tube were projected to the furnace outside each 100 mm. To deposit cobalt oxide electrocatalysts, cobalt(II) acetate tetrahydrate (Daejung, ≥98% purity) powder was used as a precursor. The precursor was placed with the amount of 10 mg within an alumina boat (50 mm in length × 10 mm in width × 9 mm in height), which was located at the center of the tube. A bare carbon fiber paper (CFP, Toray paper 120) substrate with dimensions of 2 cm in length × 1 cm in width covered the alumina boat where the deposition side of the CFP substrate faced down looking at the precursor. After an initial Ar purge for 30 min, the precursor powder was heated to 400, 500, 600, 700, or 800 °C at a ramp rate of 20 °C/min and transported under a 30 cc/min Ar flow at 1 atm. After the furnace was held at each temperature for 10 min, it was allowed to cool naturally to room temperature. To identify the sintering effect, the processing was carried out with various reaction times (10 min, 0.5 h, and 1 h) at 400 or 500 °C.

**Scheme 1:**
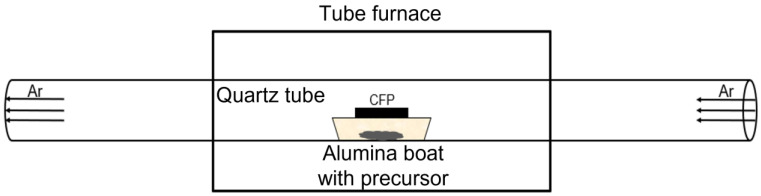
Illustration of the thermal metal–organic deposition (MOD) process.

The thermal MOD process developed in this work is illustrated in Scheme 1. In ambient Ar, since the diffusion length of depositing species is short, the CFP substrate is placed at a close distance of 9 mm from the precursor while covering the alumina boat. The deposition side of the CFP substrate faces down looking at the precursor. Such a close distance between substrate and precursor makes the deposition of nanostructured films easier. As a pretest, we put alumina substrates on both side seats outside the alumina boat to check whether there were any material species stained on the alumina substrates after the process. However, it was confirmed that there were no stains. Therefore, it could be inferred that the diffusion length of the depositing species from the precursor is short enough not to escape from the alumina boat within a given processing time frame.

### 2.2. Structural Characterization

The thermal decomposition behavior of cobalt(II) acetate tetrahydrate [(CH_3_COO)_2_Co·4H_2_O], used as a precursor for the deposition of cobalt oxide, was investigated by thermogravimetric analysis (TGA) and differential scanning calorimetry (DSC) (Discovery SDT 650, TA Instruments, Newcastle, DE, USA) from 40 to 800 °C at a scan rate of 10 °C/min using Tzero aluminum pans in ambient Ar. Phase assignment was carried out with the help of X-ray diffraction (XRD) using an X’pert Pro (Hong Kong, China) equipped with a Cu Kα source (λ = 1.5406 Å), as well as confocal Raman microprobe analysis using a Horiba XploRA instrument (Lonrimore, France). Raman spectra were collected with excitation from the 532 nm line of an air-cooled solid laser. The morphology and composition of the samples were observed by field-emission scanning electron microscopy (FE-SEM) and energy dispersive spectroscopy (EDS) using a Hitachi S-4800 instrument (Tokyo, Japan). The surface chemical state was investigated by X-ray photoelectron spectroscopy (XPS, Thermo Scientific, Waltham, MA, USA) with monochromatized Al K_α_ radiation where the energy calibration was achieved by setting the hydrocarbon C 1s line at 284.80 eV. The crystal structure was further examined by high-resolution transmission electron microscopy (HRTEM) using an FEI Titan G2 60-300 S/TEM (Bangalore, India) equipped with a Cs probe corrector (Auckland, New Zealand) and ChemiSTEM technology.

### 2.3. Electrochemical Characterization

The OER properties of the cobalt oxide electrocatalysts deposited on CFP were evaluated using a three-electrode cell (SP-200, Bio-Logic potentiostat, Knoxville, TN, USA). All measurements were performed in a 1 M aqueous solution of KOH (purity ≥ 85%) purged with N_2_ gas at room temperature. The cobalt oxide electrocatalysts deposited on CFP were individually used as the working electrodes, whereas a Pt wire and a Hg/HgO [1 M KOH, 0.098 V versus reversible hydrogen electrode (RHE)] were used as the counter and reference electrodes, respectively. The potentials were converted to the RHE standard scale by using an equation (E_RHE_ = E_Hg/HgO_ + 0.0591 pH + 0.098). Polarization curves for the OER were obtained using linear sweep voltammetry (LSV) at a scan rate of 8 mV/s, which were corrected for the ohmic potential drop (iR) losses; R is the series resistance of the electrochemical cell as determined by electrochemical impedance spectroscopy (EIS) measurements which were measured at an open circuit potential from 200 kHz to 50 mHz with an amplitude of 25 mV (Figure S1 of the Supplementary Materials). The EIS measurements for obtaining the series resistance (R_s_) and charge transfer resistance (R_ct_) values were performed at 1.68 V versus RHE. In order to estimate the electrochemically active surface area (ECSA) of the electrocatalysts, the double-layer capacitance (C_dl_) of the electrocatalysts was determined by cyclic voltammetry (CV) in the non-Faradaic potential region at different scan rates of 20–100 mV/s in 1 M KOH aqueous solution.

## 3. Results and Discussion

**Figure 1:**
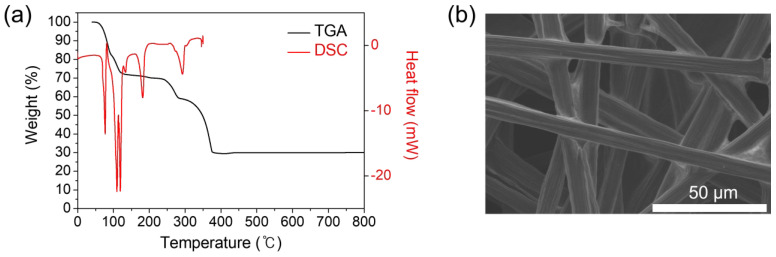
(**a**) TGA and DSC profiles of cobalt(II) acetate tetrahydrate [(CH_3_COO)_2_Co·4H_2_O], used as a precursor. (**b**) FE-SEM image showing the entire picture of a bare carbon fiber paper (CFP) substrate.

TGA and DSC curves of cobalt(II) acetate tetrahydrate [(CH_3_COO)_2_Co·4H_2_O], used as a precursor for the deposition of cobalt oxide, are shown in Figure 1a, which are acquired in ambient Ar. The initial thermal decomposition with a mass loss of ca. 30% advances to around 122 °C, relevant to the dehydration with the endothermic reactions in DSC. The dissociation of acetate groups from (CH_3_COO)_2_Co begins at around 245 °C and is completed at around 378 °C with a mass loss of ca. 40% and the additional endothermic reactions at 182 °C and 292 °C. The concomitantly generated water and acetate vapors are believed to be removed by a continuous flow of Ar. As a result, the final residue remains roughly constant at 30 wt%, which indicates the formation of cobalt oxide. The obtained cobalt oxide arises from the reaction of cobalt ions with oxygen ions dissociated from the acetate group under the decomposition of the precursor. At this point, it should be noted that the mass loss calculation results in the final residue of cobalt oxide, not cobalt metal; the melting point and boiling point of Co_3_O_4_ are 895 °C and 900 °C, respectively, and those of Co metal are 1495 °C and 2927 °C, respectively, at standard temperature and pressure (STP) [24]. Indeed, the XRD pattern of the residue left in an alumina boat after thermal MOD processing for 10 min at 400 °C confirms the direct formation of Co_3_O_4_ from the precursor in Figure S2. It is consequently found that the thermal MOD route involves the thermal evaporation and decomposition of cobalt(II) acetate tetrahydrate leading to the direct formation of cobalt oxide (Co_3_O_4_). Herein, a conductive porous CFP has been used as a substrate to facilitate electrocatalytic evaluation and to further provide a larger surface area given that the deposited film appears to conformally adhere to the underlying substrate. The CFP substrate is composed of numerous individual carbon fibers (ca. 7 μm in diameter) which are interconnected with a branch shape (Figure 1b).

**Figure 2:**
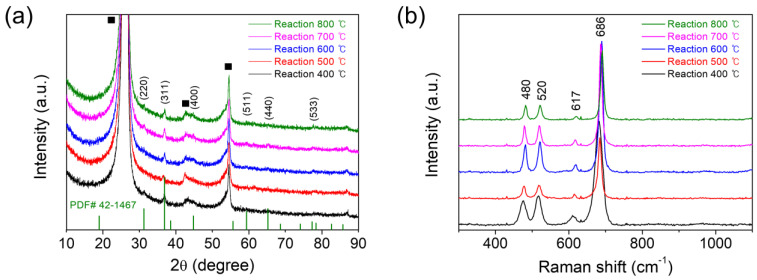
(**a**) XRD and (**b**) Raman spectra acquired for cobalt oxides (Co_3_O_4_) deposited on CFP substrates by the thermal MOD process for 10 min at 400, 500, 600, 700, or 800 °C. In (**a**), the solid squares are denoted as the peaks arising from the CFP substrates.

The MOD reaction temperatures (400–800 °C) were determined in the range between the complete decomposition temperature (378 °C) of the precursor from the earlier TGA and DSC results and the melting point of Co_3_O_4_ (895 °C at STP). Figure 2a exhibits the XRD patterns, acquired for the samples deposited on CFP substrates by the thermal MOD process for 10 min at 400, 500, 600, 700, or 800 °C. The XRD peaks for all the samples are matched well with the powder diffraction file (PDF) no. 42-1467 of the International Centre for Diffraction Data (ICDD), even if those peaks are not well distinguished because of the strong peaks arising from the CFP substrate. Such results indicate that the products are polycrystalline Co_3_O_4_. More clear phases are identified in the Raman spectra (Figure 2b). The four bands observed at 480, 520, 617, and 686 cm^−1^ are ascribed to the Raman-active modes of E_g_, F_2g_, F_2g_, and A_1g_ symmetry in Co_3_O_4_, respectively [25,26]. To identify the applicability of the thermal MOD process on the flat substrates, the additional depositions of Co_3_O_4_ have been performed on the flat substrates of SiO_2_ (2 μm-thickness)/Si and alumina (polycrystalline α-Al_2_O_3_) for 10 min at 500 °C. Figure S3 shows the XRD patterns acquired for Co_3_O_4_ deposited on SiO_2_/Si substrate, bare SiO_2_/Si substrate, and Co_3_O_4_ deposited on alumina substrate by the thermal MOD process for 10 min at 500 °C. In all cases, it is found that polycrystalline Co_3_O_4_ deposits are well formed on such flat substrates, although the peaks arising from the SiO_2_/Si and alumina substrates are detected together, indicating that this thermal MOD process can be universally applied.

**Figure 3:**
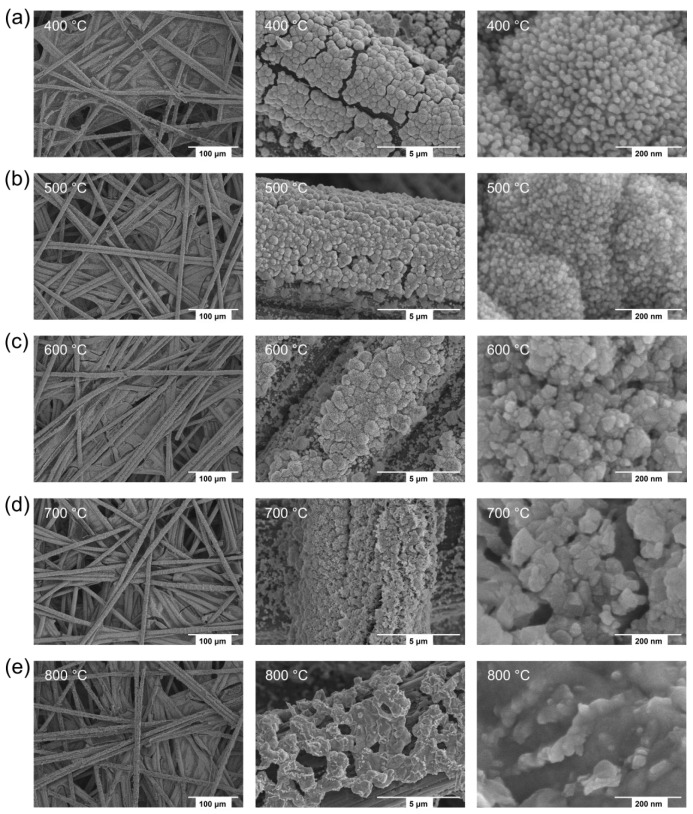
FE-SEM images acquired for cobalt oxides (Co_3_O_4_) deposited on CFP substrates by the thermal MOD process for 10 min at (**a**) 400, (**b**) 500, (**c**) 600, (**d**) 700, or (**e**) 800 °C: the low-magnification images (left) showing the entire picture of the Co_3_O_4_-deposited CFP substrates, the intermediate-magnification images (middle) focusing on the individual carbon fibers covered by the Co_3_O_4_ deposits, and the high-magnification images (right) focusing on the Co_3_O_4_ deposits.

The FE-SEM micrographs have been obtained to observe the morphologies of the Co_3_O_4_ deposits prepared on the CFP substrates by the reactions for 10 min at 400, 500, 600, 700, or 800 °C. As shown in Figure 3, the Co_3_O_4_ deposits uniformly cover the surface of individual carbon fibers (ca. 7 μm in diameter) constituting the CFP throughout the reaction temperature. The middle and right (high-magnification) images in Figure 3 show the enlarged forms of the Co_3_O_4_ deposits that cover one carbon fiber each, respectively. In particular, it is found that the small primary particles constitute the large secondary particles within the Co_3_O_4_ deposits prepared at 400, 500, and 600 °C (Figure 3a–c). The particle size is estimated to be 18.5 nm and 570 nm at 400 °C, 13.9 nm and 506 nm at 500 °C, and 27.9 nm and 780 nm at 600 °C in diameter for small (primary) and large (secondary) particles, respectively. The smaller particle sizes at 500 °C than at 400 °C are considered to be related to the increase in density and crystallinity due to the reaction at a higher temperature. The larger particle sizes at 600 °C than at 500 °C are attributed to the particle growth upon the reaction at a much higher temperature. Upon the reaction at 700 °C, the large particles collapse and only one kind of small particle (36.7 nm in diameter) exists. For the Co_3_O_4_ prepared at 800 °C, the particles are sintered and the carbon fiber surface is exposed sparsely. In Figure S4, the EDS elemental maps for the Co_3_O_4_ deposited on CFP by the thermal MOD process for 10 min at 500 °C are shown with low and high magnification images, exhibiting the high uniformity of Co_3_O_4_ deposits on CFP. Furthermore, the XPS spectra of Co 2p and O 1s, acquired for the Co_3_O_4_ deposited on CFP substrate by the thermal MOD process for 10 min at 700 °C, well show the chemical state of Co_3_O_4_ involving Co^2+^, Co^3+^, surface-adsorbed oxygen, and lattice oxygen (Figure S5).

**Figure 4:**
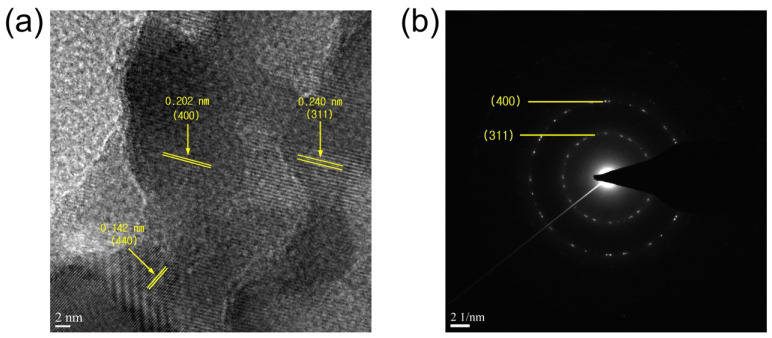
(**a**) HRTEM image and (**b**) SAED pattern acquired for the particle harvested from the cobalt oxide (Co_3_O_4_) deposited on CFP substrates by the thermal MOD process for 10 min at 700–800 °C.

For the particles harvested from the Co_3_O_4_ deposited on CFP substrates for 10 min at 700 °C, the crystal structure has been investigated by HRTEM. The typical lattice fringes of (311), (400), and (440) of Co_3_O_4_ are observed in the HRTEM image (Figure 4a) and the selected area electron diffraction (SAED) pattern (Figure 4b).

**Figure 5:**
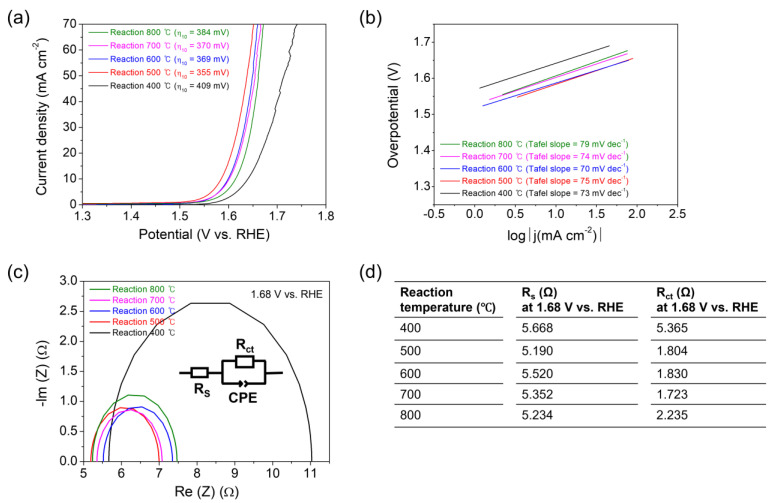
(**a**) Polarization curves and (**b**) Tafel plots measured for the Co_3_O_4_ electrocatalysts deposited on CFP substrates by the thermal MOD process for 10 min at 400, 500, 600, 700, or 800 °C. (**c**) Nyquist plots measured for each sample at 1.68 V versus RHE where the inset depicts the corresponding equivalent circuit model used to extract the series resistance (R_s_) and charge transfer resistance (R_ct_). The R_s_ and R_ct_ values measured for each sample are arranged in the table of (**d**). The data have been acquired in aqueous solutions of 1 M KOH using a three-electrode assembly.

The electrocatalytic OER properties of the Co_3_O_4_ deposits on CFP have been investigated using a three-electrode setup in 1 M KOH aqueous solution. Figure 5a,b exhibits polarization curves and Tafel plots which have been corrected for ohmic potential drop (*iR*) losses, respectively. The Co_3_O_4_ prepared at the lowest calcination temperature of 400 °C shows the highest value of 409 mV in overpotential (η_10_) required to reach a current density of 10 mA cm^−2^, whereas the others represent similar η_10_ values in the range from 355 to 384 mV (Figure 5a). Moreover, Tafel slopes are acquired at similar levels ranging from 70 to 79 mV dec^−1^ for all the samples (Figure 5b). It suggests that the OER occurs through the same reaction mechanism (or rate-determining step) on the Co_3_O_4_/CFP electrocatalysts, as a change in Tafel slope is indicative of a change in the reaction mechanism, or in rate-determining steps within the reaction pathway [27,28]. The various mechanistic schemes on the OER in alkaline solution have been proposed over the years with the honorable works of Bockris and Otagawa [29], Krasil’shchikov [30], Kobussen and Broers [31], and O’Grady and Yeager [32]. Krasil’shchikov’s OER mechanism, one of the widely known mechanisms, can be described by the following reactions with the corresponding Tafel slopes [30,33].

S + OH^−^ ⇄ SOH + e^−^ Tafel slope = 120 mV dec^−1^ (1)

SOH + OH^−^ ⇄ SO^−^ + e^−^ Tafel slope = 60 mV dec^−1^ (2)

SO^−^ → SO + e^−^ Tafel slope = 45 mV dec^−1^ (3)

2SO → 2S + O_2_ Tafel slope = 19 mV dec^−1^ (4)

where S is a catalytically active surface site. Besides those reports, the early physisorbed hydrogen peroxide model and the recent model by Lyons et al. were reliably constructed with the combination of experiments and DFT calculations [27,34]. The physisorbed hydrogen peroxide model is described by the following reaction sequence [27].

S + OH^−^ → S–OH + e^−^ (5)

S–OH + OH^−^ → S–H_2_O_2_ + e^−^ (6)

S–H_2_O_2_ + OH^−^ → S–HO_2_^−^ + H_2_O (7)

S–H_2_O_2_ + S–HO_2_^−^ → O_2_(g) + H_2_O + OH^−^ (8)

where S is a catalytically active surface site and –H_2_O_2_ represents physisorbed hydrogen peroxide. When reaction (6) is taken as the rate-determining step with an intermediate coverage of S–OH, a Tafel slope is ca. 60 mV dec^−1^ at 25 °C [27]. The recently suggested model is given below [34].

S*OH_2_ + OH^−^ → S*OH^−^ + H_2_O (9)

S^*^OH^−^ → S*OH + e^−^ (10)

S*OH + OH^−^ → S*O^−^ + H_2_O (11)

S*O^−^ → S*O + e^−^ (12)

S*O + OH^−^ → S*OOH + e^−^ (13)

S*OOH + OH^−^ → S*O_2_ + H_2_O + e^−^ (14)

S*O_2_ + OH^−^ → S*OH^−^ + O_2_ (15)

where S* represents a surfaquo group attached to the hydrous oxide surface by bridging oxygen ligands. When the reaction step (11) is rate determining, a Tafel slope is ca. 60 mV dec^−1^ at 25 °C [34]. Recently, it was reported that metal superoxide species work as an active intermediate in the rate-determining step of the OER with the release of dioxygen from the superoxide intermediate [35]. By comparing the addressed models above and the Tafel slope values (70–79 mV dec^−1^) acquired for the samples in this work, it is inferred that the OER rate-determining step corresponds to the reaction steps with the Tafel slope of 60 mV dec^−1^, despite 10–19 mV dec^−1^ greater Tafel slopes. In Table S1 of the Supplementary Materials, the OER properties of some Co_3_O_4_ electrocatalysts reported in the literature are compiled. The η_10_ and Tafel slope values of the samples prepared in this work are comparable to the previously reported values (see the references in Table S1 of the Supplementary Materials).

The Nyquist plots measured at 1.68 V versus RHE by electrochemical impedance spectroscopy (EIS) for the Co_3_O_4_ electrocatalysts are shown in Figure 5c. The plots are fitted to the equivalent circuit model (the inset in Figure 5c), which consists of the series resistance (R_s_) including the electrolyte resistance, contact resistance, and intrinsic resistance of the electrode, the charge transfer resistance (R_ct_), and the constant phase element (CPE) [12]. The R_s_ and R_ct_ values acquired for the samples prepared at the different reaction temperatures are arranged in the table of Figure 5d. The R_s_ reveals the roughly constant values ranging from 5.190 to 5.668 Ω for all the samples. The R_ct_ values are 5.365, 1.804, 1.830, 1.723, and 2.235 Ω for the samples prepared at 400, 500, 600, 700, and 800 °C, respectively. Noticeably, the R_ct_ value acquired for the Co_3_O_4_ prepared at 400 °C is the highest, which is associated with the highest η_10_. The low R_ct_ values in the Co_3_O_4_ electrocatalysts prepared at 500, 600, 700, and 800 °C are related to the low η_10_ values. Additionally, Nyquist plots measured at 1.48 and 1.58 V versus RHE are shown in Figure S6.

We distinguish the surface area and the electrochemically active surface area (ECSA), where the surface area includes both the electrochemically non-active and active surface area. For example, although the surface area is low, the inherent catalytic activity of an individual active site of the ECSA can be high. On the other hand, although the surface area is high, the inherent catalytic activity of an individual active site of the ECSA can be low. The total electrode-level electrocatalytic activity appears as a sum of them.

**Figure 6:**
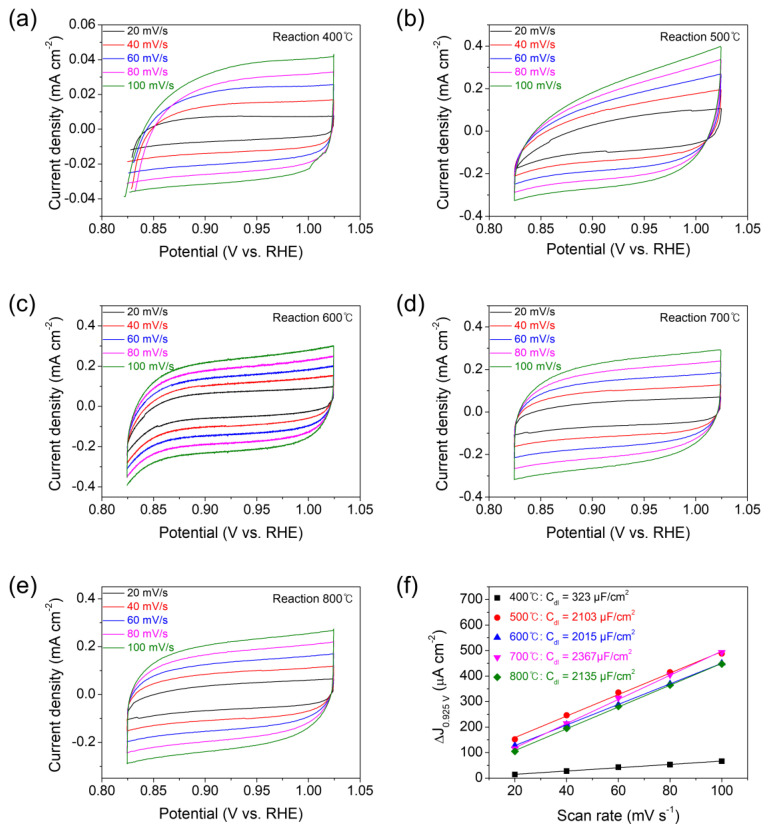
Cyclic voltammograms acquired at scan rates of 20–100 mV/s in the range of 0.825–1.025 V versus RHE for the Co_3_O_4_ electrocatalysts deposited on CFP substrates by the thermal MOD process for 10 min at (**a**) 400, (**b**) 500, (**c**) 600, (**d**) 700, or (**e**) 800 °C. (**f**) The difference (Δj) of anodic and cathodic current densities at 0.925 V versus RHE (in the non-Faradaic region) plotted as a function of the scan rate. Each plot is fitted to a straight line to determine the double-layer capacitance (C_dl_) values.

**Figure 7:**
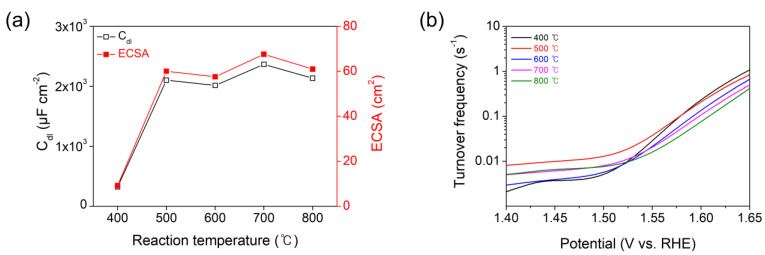
(**a**) Double-layer capacitances (C_dl_) and electrochemically active surface areas (ECSA) plotted as a function of reaction temperature for the Co_3_O_4_ electrocatalyst deposited on CFP substrate by the thermal MOD process. (**b**) Turnover frequencies (TOF) of the Co_3_O_4_ electrocatalysts.

In this regard, for further mechanistic investigation, the ECSAs of all the samples have been obtained by measuring the double-layer capacitance (C_dl_) from cyclic voltammetry (CV) data across a potential range with no Faradaic current [12,36]. The CVs have been collected at various scan rates (20−100 mV/s) in the potential range of 0.825−1.025 V versus RHE, where the current is preponderantly due to the charging of the double layer, not due to proton reduction (Figure 6a–e). The differences (Δj) of anodic and cathodic current densities at 0.925 V versus RHE for the CV plots of each sample are plotted as a function of the scan rate in Figure 6f. The slope of each Δj versus scan rate plot is equal to a value of 2C_dl_. The ECSAs have been obtained from the ratio of the measured C_dl_ with respect to the specific capacitance of flat crystalline Co_3_O_4_ (35 μF/cm^2^ in 1 M KOH) [37]. The resulting C_dl_ and ECSA values are displayed as a function of the reaction temperature in Figure 7a. The Co_3_O_4_ prepared at 400 °C exhibits the lowest values of C_dl_ (323 μF/cm^2^) and ECSA (9.22 cm^2^), which result in the highest values of R_ct_ and η_10_. The Co_3_O_4_ electrocatalysts prepared at 500, 600, 700, and 800 °C reveal the high values of C_dl_ (ca. 2000 μF/cm^2^) and ECSA (ca. 60 cm^2^) at similar levels, leading to the low values of R_ct_ and η_10_.

The turnover frequency (TOF), defined as the number of O_2_ molecules evolved per active site per unit of time, is used to assess the inherent catalytic activity of the electrocatalyst. The TOF can be calculated using Equation (16) [38].

(16) $$TOF=\frac{Total\;oxygen\;turn\;over\times j}{Active\;surface\;sites\;\left(\frac{atoms}{cm^{2}}\right)\times ECSA}$$

where *j* is the current density and ECSA is the electrochemically active surface area of the electrocatalyst. The number of total oxygen turnovers is calculated by the following Equation (17).

(17) $$*O_{2}=j\left(\frac{mA}{cm^{2}}\right)\left(\frac{1\;Csec^{-1}}{1000\;mA}\right)\left(\frac{1\;mole^{-1}}{96485.3\;C}\right)\left(\frac{1\;mol\;O_{2}}{4\;mole^{-}}\right)\left(\frac{6.023\times 10^{23}O_{2}\;molecules}{1\;molO_{2}}\right)$$

The active surface sites are calculated using Equation (18) [39].

(18) $$Active\;sites_{Co_{3}O_{4}}={\left(\frac{14\;atom/unit\;cell}{148.195\;{Å}^{3}/unit\;cell}\right)}^{\frac{2}{3}}=2.07\times 10^{15}\;cm^{-2}$$

where the atom/unit cell and the volume/unit cell of Co_3_O_4_ were brought from references [40] and [41], respectively. The resulting TOF (per active site) plots of the Co_3_O_4_ electrocatalysts in the applied potential range of 1.40 to 1.65 V versus RHE are shown in Figure 7b. The TOF values of the electrocatalysts at 1.6 V versus RHE are measured to be 0.234, 0.209, 0.136, 0.107, and 0.076 O_2_/s per active site for the Co_3_O_4_ electrocatalysts prepared at 400, 500, 600, 700, and 800 °C, respectively. In particular, it is noteworthy that the TOF decreases with increasing reaction temperature. These results highlight that the inherent catalytic activity of the surface sites of the Co_3_O_4_ prepared at 400 °C is the highest despite its poor electrode-level OER activity with the lowest ECSA, highest R_ct_, and highest η_10_. As reaction temperature increases, the crystallinity of the Co_3_O_4_ electrocatalyst is improved which, in turn, brings about the enhanced OER activity (at the electrode level) with the high ECSA, low R_ct_, and low η_10_, which is attributed to the enhanced electrical conductivity. However, the inherent catalytic activity (represented by the TOF) of the surface sites of Co_3_O_4_ decreases with reaction temperature due to the high-temperature sintering effect, as per the aforementioned FE-SEM results. Nevertheless, the similar Tafel slope values for all the samples indicate that the reaction kinetics (the rate-determining step) for the OER is equal in the Co_3_O_4_ electrocatalysts throughout the reaction temperature.

The negative impact of the sintering effect on the OER activity has been further examined. Given that sintering is a function of time [42,43], the η_10_ and Tafel slope values of the Co_3_O_4_ samples were acquired with varying reaction times (10 min, 0.5 h, and 1 h) at the reaction temperature of 400 °C (Figure S7) or 500 °C (Figure S8). Those results indicate that the OER activity of Co_3_O_4_ decreases with gradual increases in the η_10_ and Tafel slope values as reaction time increases.

**Figure 8:**
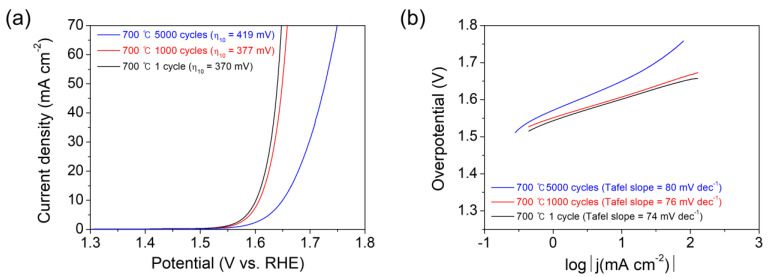
(**a**) Polarization curves and (**b**) Tafel plots of the Co_3_O_4_ electrocatalysts deposited on CFP substrates by the thermal MOD process for 10 min at 700 °C, acquired after the 1st, 1000th, or 5000th CV cycling in the range between 0.824 and 1.624 V versus RHE at a scan rate of 100 mV/s.

The stability under repeated CV sweeps has been assessed for the Co_3_O_4_ electrocatalysts prepared at 700 °C. CV sweeps have been performed for 1000 or 5000 cycles in 1 M KOH aqueous solution in the range between 0.824 V and 1.624 V versus RHE at a scan rate of 100 mV/s. The polarization curves and Tafel plots acquired for the samples after the 1st, 1000th, or 5000th cycles are shown in Figure 8a,b, respectively. The small shifts in the η_10_ (from 370 to 377 mV) and Tafel slope (from 74 to 76 mV dec^−1^) are found during 1000 cycles, indicating permissible durability. After the 5000th cycle, the η_10_ and Tafel slope increase further to 419 mV and 80 mV dec^−1^, respectively. The stability was further investigated by using the chrono-current method for 10 h under a steady current of 10 mA, as shown in Figure S9. In the time regime, a constant potential at 1.7 V versus RHE was maintained, verifying the stability of the catalyst. The XPS spectra of Co 2p and O 1s acquired for the Co_3_O_4_ electrocatalyst after the 5000th CV cycling (Figure S10) indicated that the Co^2+^ component is less and adsorbed oxygen (hydroxyl group) component is more in the surface region of the electrocatalyst after the CV cycling compared to before the CV cycling (Figure S5). Such surface modification might cause activity degradation while the electrocatalyst works.

Consequently, the results indicate that superior Co_3_O_4_ electrocatalysts are prepared under the processing conditions of optimal temperature and time. Similarly, it was reported that the electrochemical performance of a hydrothermally synthesized Co_3_O_4_-based supercapacitor is dependent on processing temperature and concomitant crystal growth and morphology, showing the best performance at optimized temperatures [44]. This work elucidates the processing–property relationship in the Co_3_O_4_ electrocatalyst by investigating the detailed OER activity systematically. Additionally, it is found that the structure and electrocatalytic activity of catalysts can be controlled facilely by modulating the processing conditions. This study thus offers the groundwork for the high-throughput fabrication and rational design of high-performance electrocatalysts.

## 4. Conclusions

The thermal MOD process was developed for the high-throughput deposition of the nanostructured Co_3_O_4_ electrocatalysts on carbon fibers (ca. 7 μm in diameter). The reaction temperature was determined in the range from 400 to 800 °C by the TGA and DSC analyses. Furthermore, the reaction time at each temperature was set for 10 min in order to minimize the sintering effect. The XRD and Raman spectroscopy results indicated that the deposits possess the polycrystalline structure of spinel-type Co_3_O_4_. The FE-SEM micrographs showed that the different microstructures evolved depending on the reaction temperature in the Co_3_O_4_ deposits uniformly covering the surface of individual carbon fibers constituting the CFP. In particular, it was found that the small primary particles constitute the large secondary particles within the Co_3_O_4_ deposits prepared at 400, 500, and 600 °C, where those particles exhibited an increase in density and crystallinity and coarsened with increasing the reaction temperature. Upon the reaction at 700 °C, the large particles collapsed, leaving only small particles behind. For the Co_3_O_4_ prepared at 800 °C, the particles were sintered with the sparsely exposed surfaces of the carbon fibers. The OER properties of the Co_3_O_4_ electrocatalysts deposited on CFP were contrasted as a function of the reaction temperature in 1 M KOH aqueous solution. For the electrocatalysts prepared at 500 °C and over, similar η_10_ values (355–384 mV) were obtained. For all the samples, Tafel slopes were acquired at similar levels ranging from 70 to 79 mV dec^−1^, suggesting that the OER occurred through the same reaction mechanism (or rate-determining step). The OER activity was estimated by the reported values in the literature and the several known mechanisms. The acquired η_10_ values were matched well with the R_ct_ values measured by the EIS. The C_dl_ and ECSA values were obtained by the CV measurements, and the TOFs were calculated for all the electrocatalysts. Those results highlighted that the improved crystallinity of the Co_3_O_4_ electrocatalyst with the increased reaction temperature leads to enhancement in electrode-level OER activity with the high ECSA, low R_ct_, and low η_10_, due to the enhanced electrical conductivity. On the other hand, it was found that the inherent catalytic activity (represented by the TOF) of the surface sites of the Co_3_O_4_ decreases with reaction temperature due to the high-temperature sintering effect.

## Data Availability

Not applicable.

## Supplementary Materials

The following supporting information can be downloaded at: https://www.mdpi.com/article/10.3390/nano13061021/s1, Table S1: The reported OER properties of Co_3_O_4_ electrocatalysts in literature; Figure S1: Nyquist plots of the Co_3_O_4_ electrocatalysts; Figure S2: XRD pattern of the residue left in an alumina boat after thermal MOD processing; Figure S3: XRD patterns acquired for Co_3_O_4_ deposited on SiO_2_/Si, alumina, and bare SiO_2_/Si substrates; Figure S4: FE-SEM images and EDS elemental maps for the Co_3_O_4_ deposited on CFP; Figure S5: XPS spectra acquired for the Co_3_O_4_ electrocatalyst; Figure S6: Nyquist plots measured at 1.48 and 1.58 V versus RHE for the Co_3_O_4_ electrocatalysts; Figure S7: Polarization curves and Tafel plots measured for the Co_3_O_4_ electrocatalysts deposited for 10 min, 0.5 h, or 1 h at 400 °C; Figure S8: Polarization curves and Tafel plots measured for the Co_3_O_4_ electrocatalysts deposited for 10 min, 0.5 h, or 1 h at 500 °C; Figure S9: Potential-time curve acquired for the Co_3_O_4_ electrocatalyst; Figure S10: XPS spectra acquired for the Co_3_O_4_ electrocatalyst after the 5000th CV cycling. References [45,46,47,48,49,50,51,52,53,54,55,56,57] are cited in the Supplementary Materials.
