# Supplementary material for: Electrocatalytic Properties of Co3O4 Prepared on Carbon Fibers by Thermal Metal–Organic Deposition for the Oxygen Evolution Reaction in Alkaline Water Electrolysis
Source: Nanomaterials (Basel). 2023 Mar 12;13(6):1021. doi: 10.3390/nano13061021 (PMC10058751; doi:10.3390/nano13061021)
Supplement: Supplementary file 1 [file nanomaterials-13-01021-s001.zip › nanomaterials-2220090-supplementary.pdf]

Supplementary Material

Electrocatalytic Properties of Co_3O_4 Prepared on Carbon Fibers by Thermal Metal–Organic Deposition for the Oxygen Evolution Reaction in Alkaline Water Electrolysis

Myeong Gyu Kim and Yun-Hyuk Choi *

School of Advanced Materials and Chemical Engineering, Daegu Catholic University, Gyeongsan 38430, Republic of Korea

*Correspondence: yunhyukchoi@cu.ac.kr

	Synthetic method	Electrolyte	η_{10} (mV)	Tafel slope (mV dec^{-1})	Reference
Co_3O_4	Chemical bath deposition	1 M KOH	315	58.7	[S1]
Co_3O_4	Hydrothermal	1 M KOH	440	43	[S2]
Co_3O_4	Spin coating	1 M NaOH	377	58	[S3]
Co_3O_4	Calcination	0.1 M KOH	450	89	[S4]
Co_3O_4	Thermal decomposition	1 M KOH	320	101	[S5]
Co_3O_4	Microwave assisted	1 M KOH	340	47	[S6]
Co_3O_4	Co-precipitation	1 M KOH	601	66	[S7]
Co_3O_4	Solvothermal	1 M KOH	497	N/A	[S8]
Bulk Co_3O_4	Nanocasting	0.1 M KOH	528	N/A	[S9]
Mesoporous Co_3O_4	Nanocasting	0.1 M KOH	411	80	[S10]
α-$Co(OH)_2Cl$	Electrochemical etching process	1 M KOH	320	53	[S11]
γ-CoOOH NS	Oxidation and exfoliation	1 M KOH	300	38	[S12]
$Co(OH)_2$	Electrodeposition	1 M KOH	290	91	[S13]
Co_3O_4			340	102	

Table S1. The reported OER properties of Co_3O_4 electrocatalysts in literature.

References

[S1] S. Hung, C. Tung, T. Chan, H. Chen, In situ morphological transformation and investigation of electrocatalytic properties of cobalt oxide nanostructures toward oxygen evolution, *CrystEngComm* **18** (2016) 6008-6012.

[S2] X. Liu, Z. Chang, L. Luo, T. Xu, X. Lei, J. Liu, X. Sun, Hierarchical $Zn_xCo_{3-x}O_4$ nanoarrays with high activity for electrocatalytic oxygen evolution, *Chem. Mater.* **26** (2014) 1889-1895.

[S3] H. Jeon, M. Jee, H. Kim, S. Ahn, Y. Hwang, B. Min, Simple chemical solution deposition of Co_3O_4 thin film electrocatalyst for oxygen evolution reaction, *ACS Appl. Mater. Interfaces* **7** (2015) 24550-24555.

[S4] Y. Liu, G. Han, X. Li, B. Dong, X. Shang, W. Hu, Y. Chai, Y. Liu, C. Liu, A facile synthesis of reduced Co_3O_4 nanoparticles with enhanced electrocatalytic activity for oxygen evolution, *Int. J. Hydrogen Energy* **41** (2016) 12976-12982.

[S5] S. Du, Z. Ren, J. Zhang, J. Wu, W. Xi, J. Zhu, H. Fu, Co_3O_4 nanocrystal ink printed on carbon fiber paper as a large-area electrode for electrochemical water splitting, *Chem. Commun.* **51** (2015) 8066-8069.

[S6] L. Li, T. Tian, J. Jiang, L. Ai, Hierarchically porous Co_3O_4 architectures with honeycomb-like structures for efficient oxygen generation from electrochemical water splitting, *J. Power Sources* **294** (2015) 103-111.

[S7] B. Lal, N. K. Singh, S. Samuel, R. N. Singh, Electrocatalytic properties of $Cu_xCo_{3-x}O_4$ ($0 \leq x \leq 1$) obtained by a precipitation method for oxygen evolution, *J. New Mat. Electrochem. Systems* **2** (1999) 59-64.

[S8] N. H. Chou, P. N. Ross, A. T. Bell, T. D. Tilley, Comparison of cobalt-based nanoparticles as electrocatalysts for water oxidation, *ChemSusChem* **4** (2011) 1566-1569.

[S9] T. Grewe, X. Deng, C. Weidenthaler, F. Schuth, H. Tuysuz, Design of ordered mesoporous composite materials and their electrocatalytic activities for water oxidation, *Chem. Mater.* **25** (2013) 4926-4935.

[S10] Y. J. Sa, K. Kwon, J. Y. Cheon, F. Kleitz, S. H. Joo, Ordered Mesoporous Co_3O_4 spinels as stable, bifunctional, noble metal-free oxygen electrocatalysts, *J. Mater. Chem. A* **1** (2013) 9992-10001.

[S11] P. F. Liu, S. Yang, L. R. Zheng, B. Zhang, H. G. Yang, Electrochemical etching of α-cobalt hydroxide for improvement of oxygen evolution reaction, *J. Mater. Chem. A* **4** (2016) 9578-9584.

[S12] J. Huang, J. Chen, T. Yao, J. He, S. Jiang, Z. Sun, Q. Liu, W. Cheng, F. Hu, Y. Jiang, Z. Pan, S. Wei, CoOOH nanosheets with high mass activity for water oxidation, *Angew. Chem. Int. Ed.* **54** (2015) 8722-8727.

[S13] P. T. Babar, A. C. Lokhande, B. S. Pawar, M. G. Gang, E. Jo, C. Go, M. P. Suryawanshi, S. M. Pawar, J. H. Kim, Electrocatalytic performance evaluation of cobalt hydroxide and cobalt oxide thin films for oxygen evolution reaction, *Appl. Surf. Sci.* **427 Part A** (2018) 253-259.

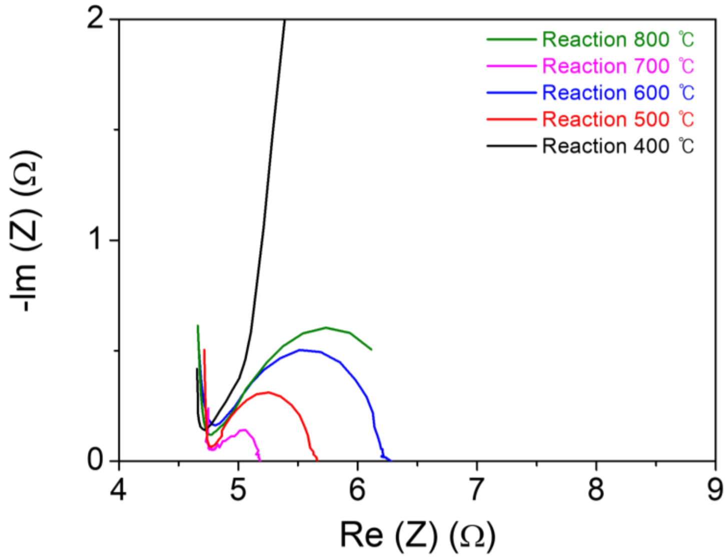

Figure S1. Nyquist plots of the Co_3O_4 electrocatalysts deposited on CFP substrates by the thermal MOD process for 10 min at 400, 500, 600, 700, or 800 °C. The plots have been acquired at an open circuit potential from 200 kHz to 50 mHz using an AC amplitude of 25 mV. The series resistance components (R_s) were measured to be 4.72, 4.77, 4.80, 4.77, and 4.77 Ω for the samples prepared at 400, 500, 600, 700, and 800 °C, respectively. Using these R_s values, the electrochemical overpotentials (η) measured in this work have been corrected by subtracting the ohmic drop (iR_s) as per: $\eta_{corr} = \eta - iR_s$.

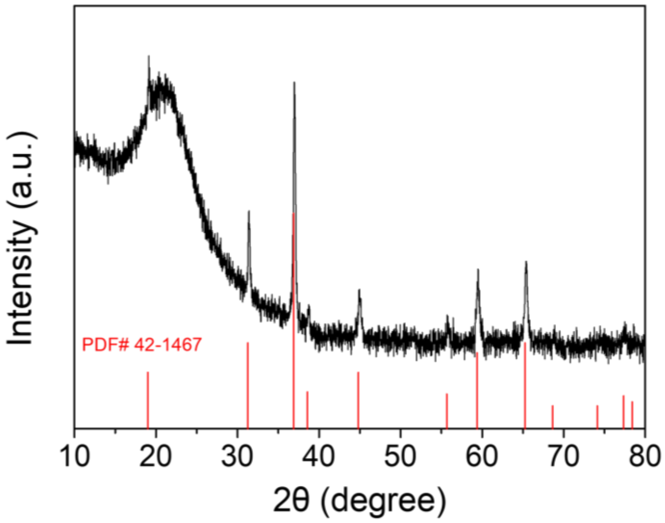

Figure S2. XRD pattern of the residue left in an alumina boat after thermal MOD processing for 10 min at 400 °C. The bottom bars are ICDD standards for Co_3O_4.

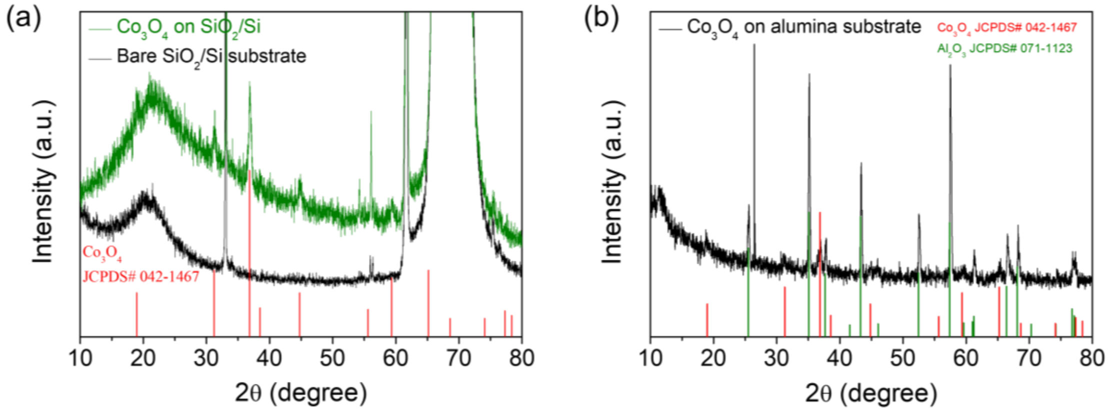

Figure S3. XRD patterns acquired for (a) Co_3O_4 deposited on SiO_2/Si substrate, bare SiO_2/Si substrate, and (b) Co_3O_4 deposited on alumina substrate by the thermal MOD process for 10 min at 500 °C. The bottom bars are ICDD standards for Co_3O_4 and Al_2O_3.

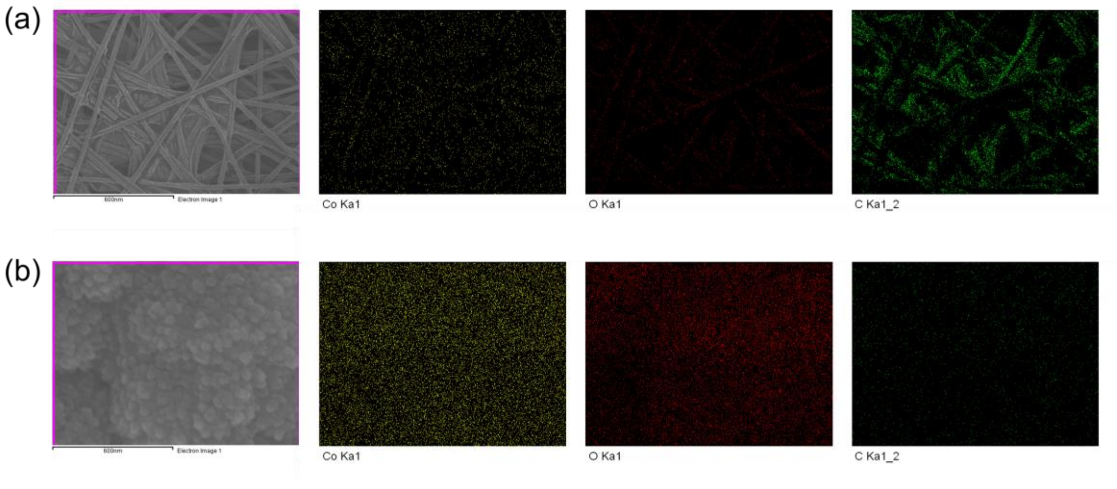

Figure S4. FE-SEM images and EDS elemental maps of Co, O, and C for the Co_3O_4 deposited on CFP by the thermal MOD process for 10 min at 500 °C. (a) Low-magnification and (b) high-magnification images.

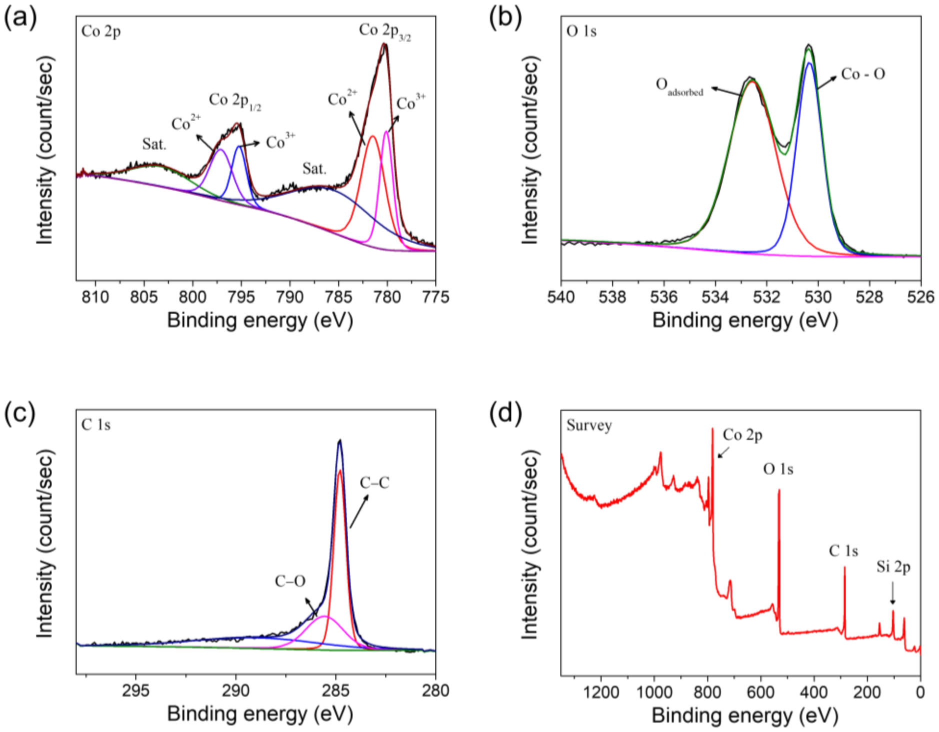

Figure S5. XPS spectra of (a) Co 2p, (b) O 1s, (c) C 1s, and (d) survey acquired for the Co_3O_4 electrocatalyst deposited on CFP substrate by the thermal MOD process for 10 min at 700 °C.

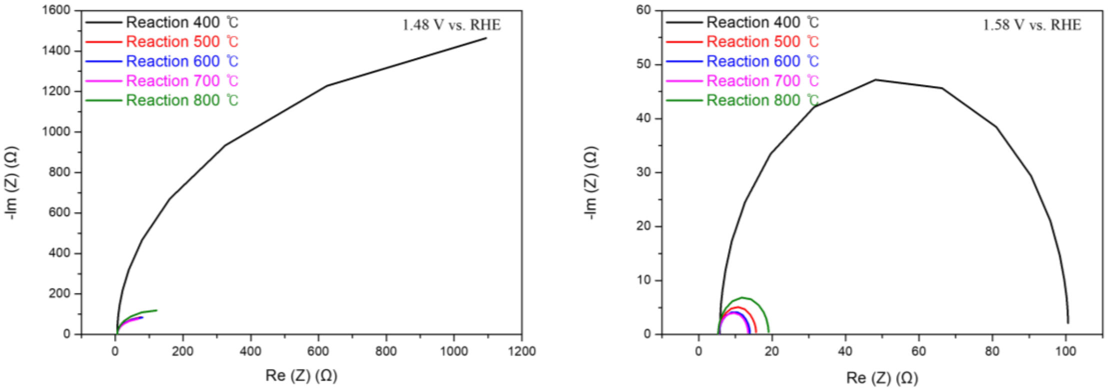

Figure S6. Nyquist plots measured at 1.48 and 1.58 V versus RHE for the Co_3O_4 electrocatalysts deposited on CFP substrates by the thermal MOD process for 10 min at 400, 500, 600, 700, or 800 °C.

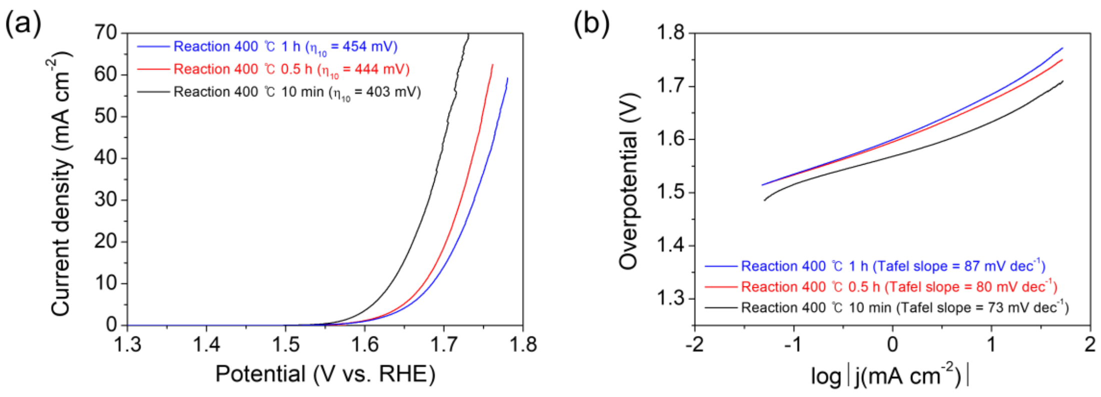

Figure S7. (a) Polarization curves and (b) Tafel plots measured for the Co_3O_4 electrocatalysts deposited on CFP substrates by the thermal MOD process for 10 min, 0.5 h, or 1 h at 400 °C.

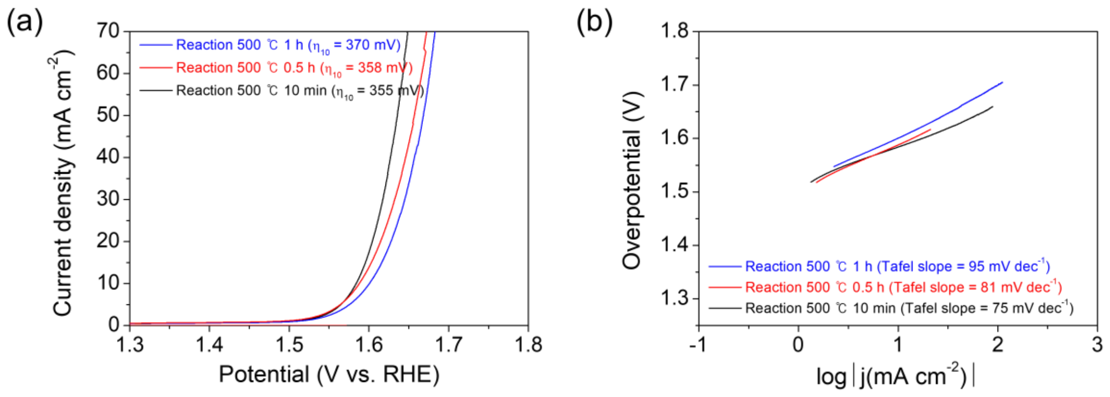

Figure S8. (a) Polarization curves and (b) Tafel plots measured for the Co_3O_4 electrocatalysts deposited on CFP substrates by the thermal MOD process for 10 min, 0.5 h, or 1 h at 500 °C.

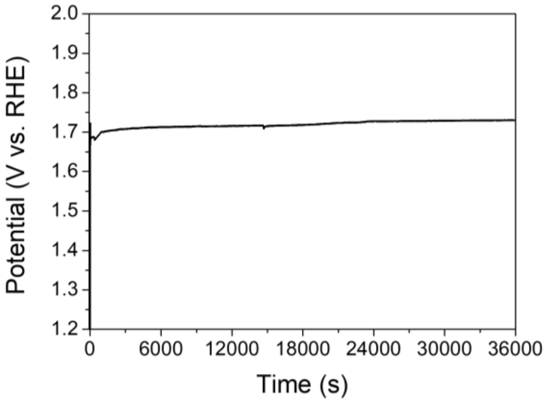

Figure S9. Potential-time curve acquired for the Co_3O_4 electrocatalyst (electrode area = 1 cm^2) deposited on CFP substrate by the thermal MOD process for 10 min at 700 °C, using the chrono-current method for 10 h under steady current of 10 mA.

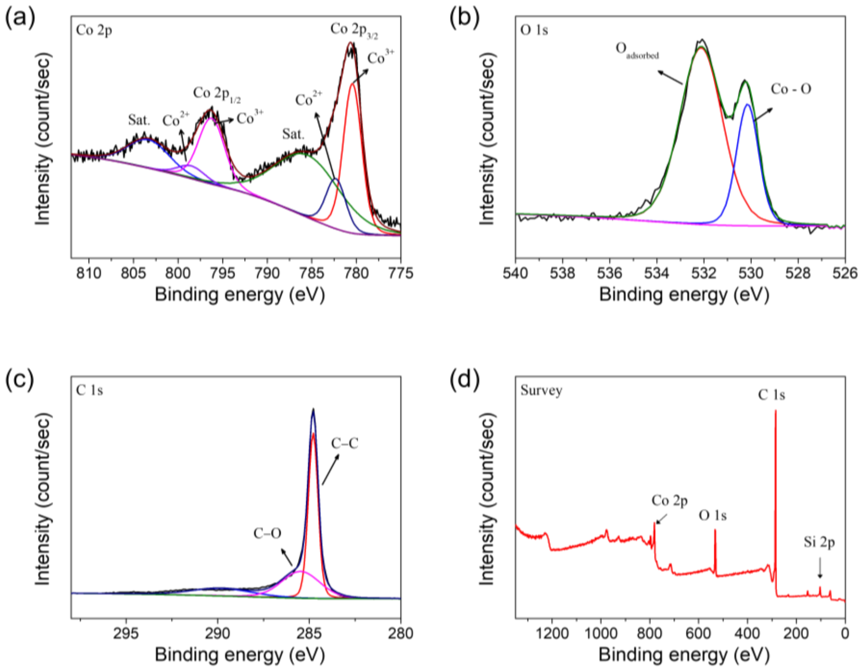

Figure S10. XPS spectra of (a) Co 2p, (b) O 1s, (c) C 1s, and (d) survey acquired for the Co_3O_4 electrocatalyst deposited on CFP substrate by the thermal MOD process for 10 min at 700 °C, after the 5000th CV cycling in the range between 0.824 and 1.624 V *versus* RHE at a scan rate of 100 mV/s.
